# Supplementary material for: The Relevance of Physico-Chemical Properties and Protein Corona for Evaluation of Nanoparticles Immunotoxicity—In Vitro Correlation Analysis on THP-1 Macrophages
Source: Int J Mol Sci. 2022 May 31;23(11):6197. doi: 10.3390/ijms23116197 (PMC9181693; doi:10.3390/ijms23116197)
Supplement: Supplementary file 1 [file ijms-23-06197-s001.zip › Supplementary material_accepted.pdf]

Supplementary material

TITLE: The Relevance of Physico-Chemical Properties and Protein Corona for Evaluation of Nanoparticles Immunotoxicity—In Vitro Correlation Analysis on THP-1 Macrophages

Mojca Pavlin [1,2,*,†], Jasna Lojk [2,†], Klemen Strojan [2], Iva Hafner-Bratkovič [3,4], Roman Jerala [3,4], Adrijana Leonardi [5], Igor Križaj [5], Nataša Drnovšek [6], Saša Novak [6], Peter Veranič [7] and Vladimir Boštjan Bregar [2]

***Corresponding author mojca.pavlin@mf.uni-lj.si**

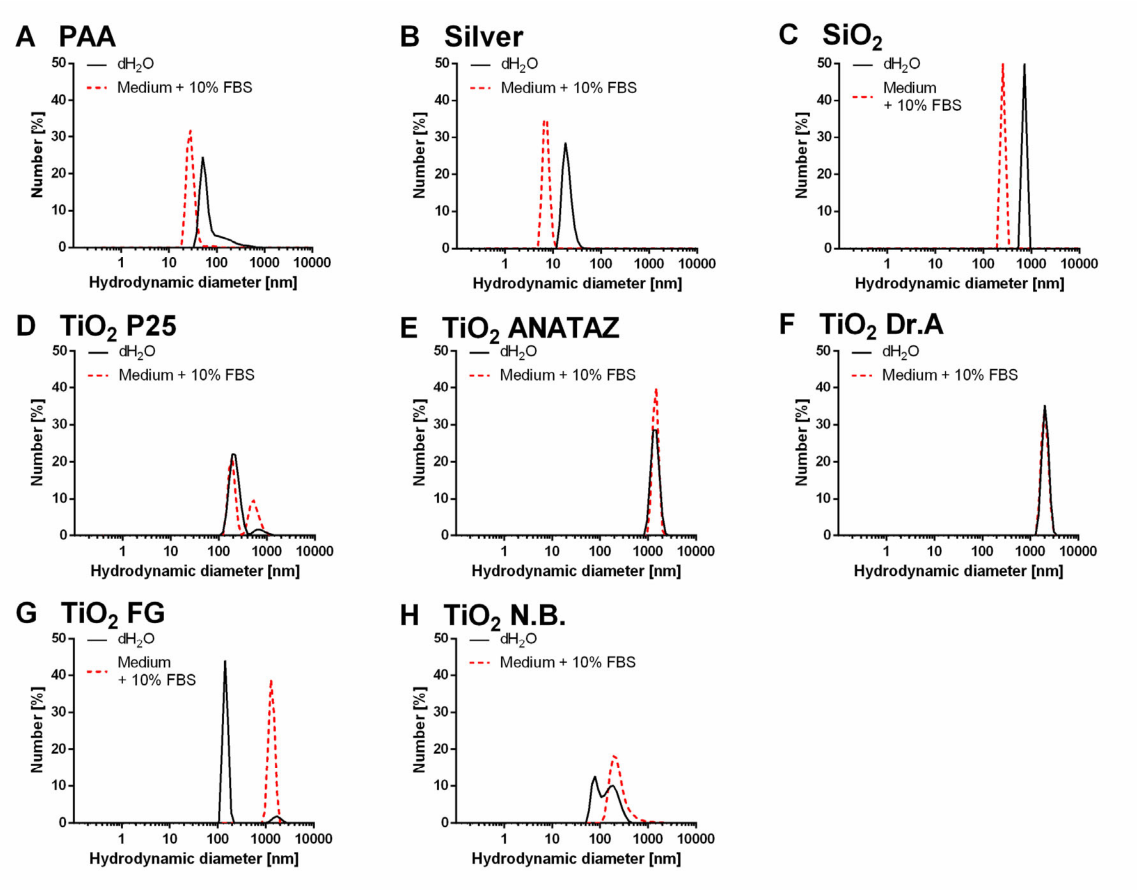

Figure S1: Size distribution by number (hydrodynamic diameter) of NPs measured by DLS in water and in complete RPMI media with 10% FBS.

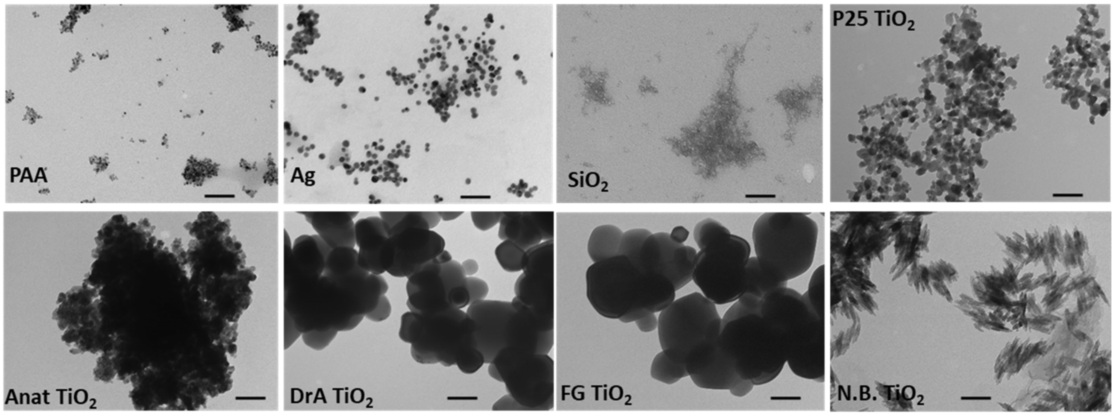

Figure S2: Transmission electron microscopy of nanoparticles dispersed in water. TEM micrographs of different NPs suspensions are shown. Scale bar: 100 nm.

Table S1: List of proteins identified by LC-ESI-MS/MS in the protein corona of the examined NPs.

**SwissProt entry name**	**Protein name**	**pI**	**Molecular mass [Da]**	**Biological process**	**% similarity with human ortholog**
A1AT	α1-antiproteinase	5.98	43694	proteinase inhibition	69/83
ALBU	serum albumin	5,60	66433	transport	76/87
ANT3	antithrombin-III	7.01	52347	haemostasis	87/92
APOA1	apolipoprotein A-I	5,36	27549	transport	78/86
APOE	apolipoprotein E	5.55	35980	cholesterol biosynthetic process	72/83
CFAH	complement factor H	6.33	138260	immune system	62/76
FETUA	α-2-HS-glycoprotein	5.26	36353	immune system	64/74
FINC	fibronectin	5.32	272153	acute-phase response	94/97
HBA	hemoglobin subunit α	8.19	15053	transport	88/92
HBBF	hemoglobin fetal subunit β	6.51	15859	transport	78/89
HS90A	heat shock protein HSP 90-α	4.92	84731	cellular response to heat	99/100
IPSP	plasma serine protease inhibitor	9,40	42495	haemostasis	71/84
TETN	tetranectin	5.47	22144	bone mineralization	85/90
THRB	prothrombin	5.97	70506	haemostasis, acute-phase response	81/89
TSP1	thrombospondin-1	4.74	129534	behavioral response to pain	97/98

*The comparison of sequences between bovine and human proteins Clustal Omega multiple sequence alignment program was used.

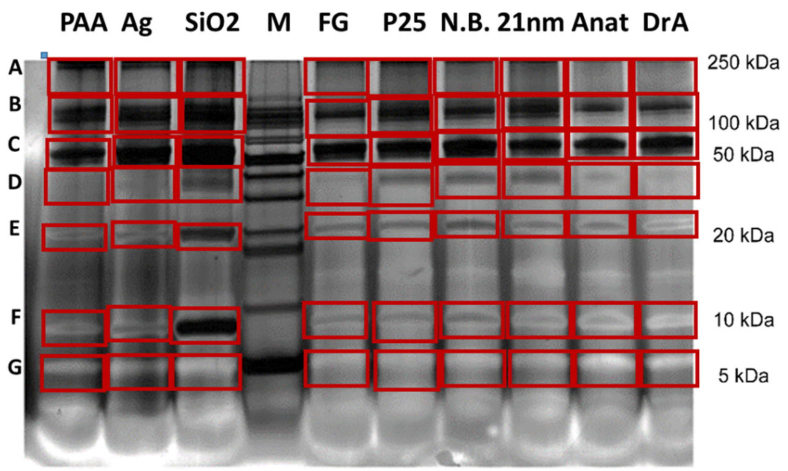

Figure S3 Analysis of NPs protein corona using SDS-PAGE of polyacrylic acid coated NPs (PAA), silver NPs (Ag), SiO2, TiO2 food-grade (FG), TiO2 P25 (P25), TiO2 N.B. (N.B.), TiO2 21nm (21 nm), TiO2 ANATAZ (Anat) and TiO2 DrA NPs. Please note that additional NPs denoted as TiO2 21nm were added as a pilot test of another bach of P25 TiO2. The bands A-G(mobbed red) were excised and analysed by MS (see Table S2). M denotes lanes loaded with molecular mass standards.

N1

protein	NC	PAA	Silver	SiO2	TiO2 P25	TiO2 ANAT	TiO2 DrA	TiO2 FG	TiO2 NB
A1AT	0	9	0	10	0	1	1	0	6
ALBU	17	340	145	153	121	109	174	400	96
ANT3	0	0	16	4	5	0	0	0	1
APOA1	4	25	1	0	1	0	9	4	15
APOE	0	36	0	0	64	0	0	4	32
CFAH	0	0	0	0	1	0	0	0	0
FETUA	1	49	25	20	9	4	5	53	24
FINC	0	0	0	0	0	16	4	0	0
HBA	1	4	1	0	1	0	9	0	1
HBBF	0	4	1	2	2	0	0	0	0
HS90A	0	0	0	0	0	0	0	0	0
IPSP	0	5	0	0	0	0	0	0	0
TETN	0	9	0	0	64	0	0	9	2
THRB	0	1	0	1	0	0	0	0	1
TSP1	0	4	0	0	0	0	0	0	0

N2

protein	NC	PAA	Silver	SiO2	TiO2 P25	TiO2 ANAT	TiO2 DrA	TiO2 FG	TiO2 NB
A1AT	0	4	3	0	3	0	0	0	0
ALBU	74	116	124	161	33	25	14	99	11
ANT3	0	0	14	0	18	2	0	0	0
APOA1	4	39	33	39	4	0	3	12	1
APOE	0	16	0	5	20	0	0	0	2
CFAH	0	0	0	0	3	0	0	0	0
FETUA	4	3	14	16	3	2	4	4	0
FINC	0	0	0	0	0	0	0	0	0
HBA	0	0	0	0	0	2	0	0	0
HBBF	0	18	5	0	0	2	0	0	0
HS90A	0	0	0	0	0	0	0	0	0
IPSP	0	0	0	0	0	0	0	0	0
TETN	0	0	0	0	0	0	0	0	0
THRB	0	0	12	0	4	0	0	0	0
TSP1	0	0	0	0	0	0	0	0	0

N3

protein	NC	PAA	Silver	SiO2	TiO2 P25	TiO2 ANAT	TiO2 DrA	TiO2 FG	TiO2 NB
A1AT	3	4	15	22	3	0	68	7	0
ALBU	193	22	166	192	86	32	562	202	155
ANT3	0	3	4	5	3	0	18	0	0
APOA1	4	12	0	56	3	0	0	0	0
APOE	0	0	0	14	4	0	0	4	0
CFAH	0	0	0	0	0	0	0	0	0
FETUA	16	0	14	4	4	6	18	5	16
FINC	0	0	0	0	0	0	0	5	0
HBA	0	3	0	19	0	0	0	0	0
HBBF	0	0	3	80	0	0	39	0	0
HS90A	0	0	0	0	5	0	4	0	0
IPSP	0	0	0	0	0	0	0	0	0
TETN	0	0	0	0	0	0	0	0	0
THRB	0	0	0	3	0	0	0	0	0
TSP1	0	0	0	0	4	0	39	0	0

Figure S4 Heatmaps of the protein abundance (spectral counts obtained from MS) in protein corona of NPs for three independent repeats (N1, N2, N3). Full names of the proteins are provided in Table S1.

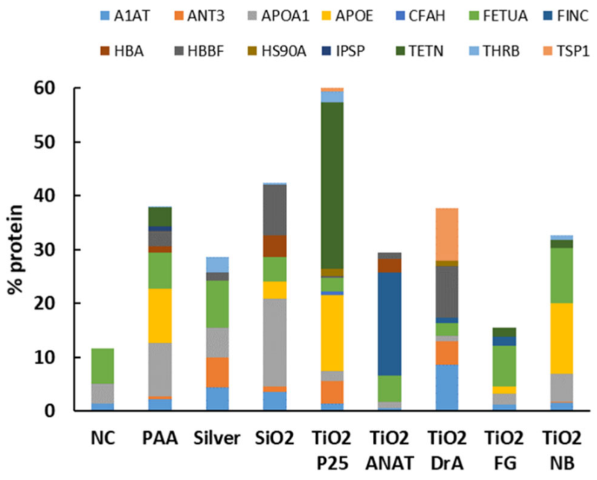

Figure S5: Relative protein abundance of proteins without albumin in the protein corona of NPs after 24h of incubation in complete cell culture medium RPMI-1640 with 10% FBS. Proteins were separated from NPs, analysed by SDS-PAGE and identified by LC-ESI-MS/MS. Spectral counts were used as a quantitative measure of the individual proteins in a sample.

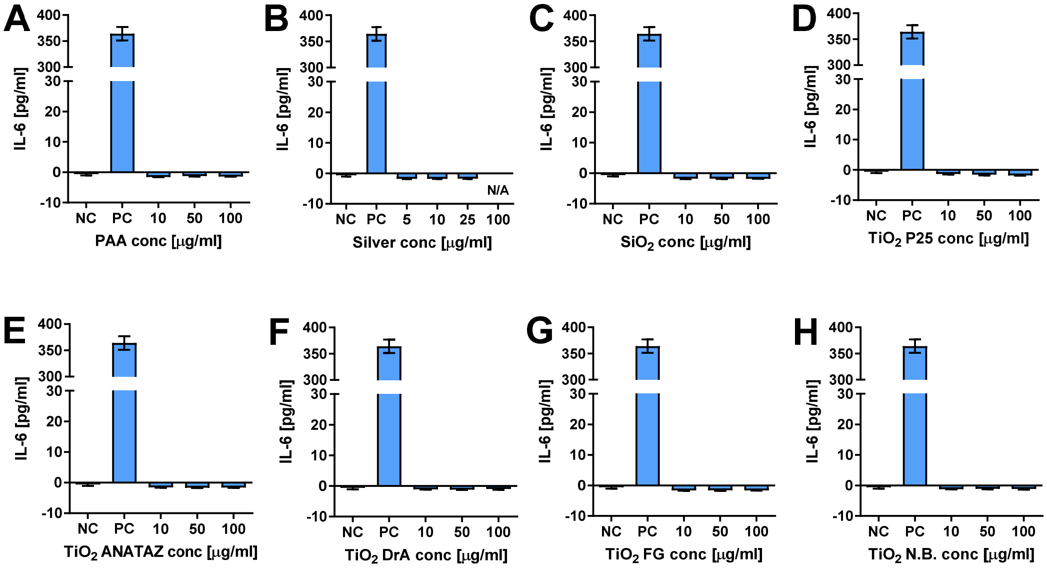

Figure S6: IL-6 secretion after 24 h incubation with increasing concentrations of (A) polyacrylic acid (PAA)-coated NPs, (B) Ag NPs, (C) industrial SiO_2 NPs, (D) TiO_2 P25 NPs, (E) TiO_2 ANATAZ NPs, (F) TiO_2 DrA NPs, (G) TiO_2 food-grade (FG) and (H) TiO_2 N.B. NPs, determined by ELISA. 1000 ng/ml LPS was used as a positive control. Shown is one of three experiments with similar results. Mean and standard error of three repeats of the same biological replicate are shown.

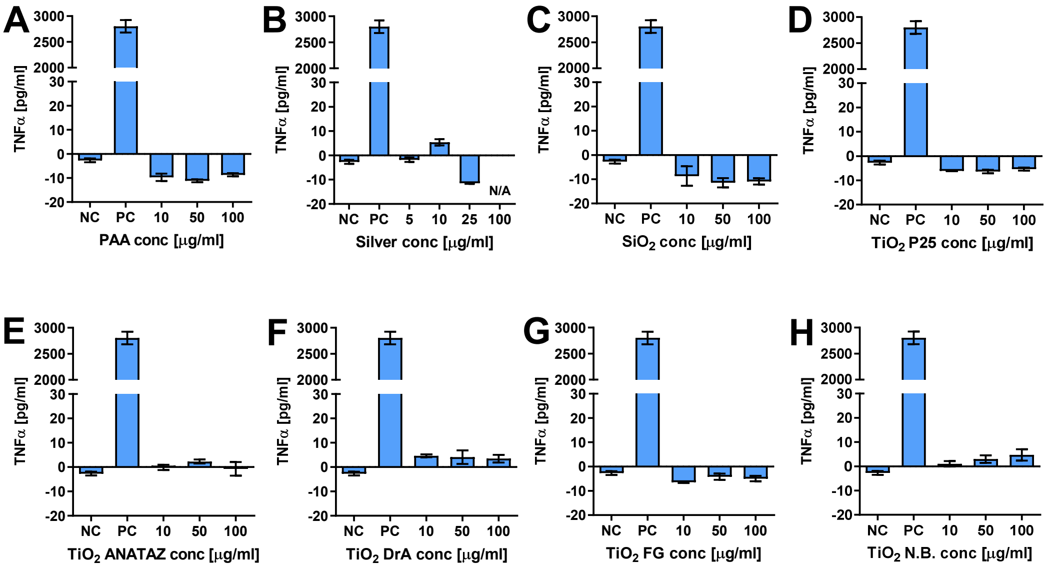

Figure S7: TNFα secretion following 24 h incubation with increasing concentration of (A) polyacrylic acid (PAA)-coated NPs, (B) silver NPs, (C) industrial SiO_2 NPs, (D) TiO_2 P25 NPs, (E) TiO_2 ANATAZ NPs, (F) TiO_2 DrA NPs, (G) TiO_2 food-grade (FG) and (H) TiO_2 N.B. NPs, determined by ELISA. 1000 ng/ml LPS was used as a positive control.

Shown is one of three experiments with similar results. Mean and standard error of three repeats of the same biological replicate are shown.

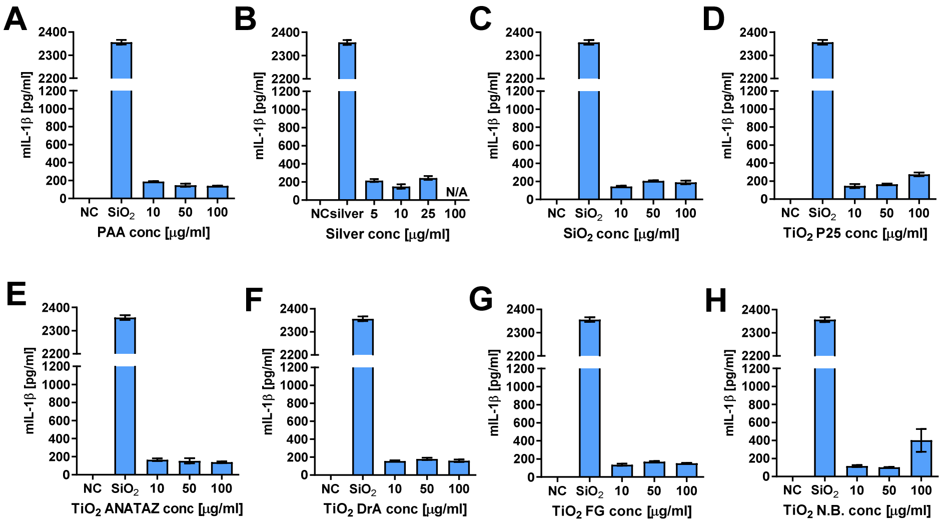

Figure S8: NP induced secretion of IL-1β in immortalized bone marrow-derived mouse macrophage cells. Cells were incubated with increasing concentration of (A) polyacrylic acid (PAA)-coated NPs, (B) silver NPs, (C) industrial SiO_2 NPs, (D) TiO_2 21 nm NPs, (E) TiO_2 ANATAZ NPs, (F) TiO_2 DrA NPs, (G) TiO_2 food-grade (FG) NPs and (H) TiO_2 N.B. NPs, determined by ELISA. 100 µg/ml crystalline SiO_2 NPs was used as a positive control. Shown is one of three experiments with similar results. Mean and standard error of three repeats of the same biological replicate are shown.

Table S2: Identification of nanoparticle corona proteins separated by SDS-PAGE (Figure S3) using the ESI-MS/MS. Three independent experiments were performed. Abbreviation: m, oxidised methionine.

See supplementary file »**Table S2-MS.xlsx**«
